# Clinical characteristics, treatment, and outcome of patients with large cell neuroendocrine carcinoma of the lung and brain metastases – data from a tertiary care center

**DOI:** 10.1007/s10585-023-10250-6

**Published:** 2023-12-08

**Authors:** Petar Popov, Ariane Steindl, Ladislaia Wolff, Elisabeth S. Bergen, Franziska Eckert, Josa M Frischer, Georg Widhalm, Thorsten Fuereder, Markus Raderer, Anna S. Berghoff, Matthias Preusser, Barbara Kiesewetter

**Affiliations:** 1 Department of Medicine I, Division of Oncology, Medical University of Vienna, Waehringer Guertel 18 – 20, Vienna, A-1090 Austria; 2 Department of Radiation Oncology, Medical University of Vienna, Vienna, Austria; 3 Department of Neurosurgery, Medical University of Vienna, Vienna, Austria

**Keywords:** Large cell neuroendocrine carcinoma of the lung, Lung cancer, Neuroendocrine carcinoma, Brain metastases

## Abstract

Large cell neuroendocrine carcinoma (LCNEC) of the lung is an aggressive malignancy, with brain metastases (BM) occurring in approximately 20% of cases. There are currently no therapy guidelines for this population as only few data on the management of LCNEC and BM have been published. For this retrospective single center study, patients with LCNEC and BM were identified from the Vienna Brain Metastasis Registry. Data on clinicopathological features, BM-specific characteristics, treatment, and outcome were extracted. In total, 52/6083 (0.09%) patients in the dataset had a diagnosis of LCNEC and radiologically verified BM. Median age at diagnosis of LCNEC and BM was 59.1 and 60.1 years, respectively. Twenty-seven (51.9%) presented with single BM, while 12 (23%) exhibited > 3 BM initially. Neurologic symptoms due to BM were present in n = 40 (76.9%), encompassing neurologic deficits (n = 24), increased intracranial pressure (n = 18), and seizures (n = 6). Initial treatment of BM was resection (n = 13), whole brain radiation therapy (n = 19), and/or stereotactic radiosurgery (n = 25). Median overall survival (mOS) from LCNEC diagnosis was 16 months, and mOS after BM diagnosis was 7 months. Patients with synchronous BM had reduced mOS from LCNEC diagnosis versus patients with metachronous BM (11 versus 27 months, p = 0.003). Median OS after BM diagnosis did not differ between LCNEC patients and a control group of small cell lung cancer patients with BM (7 versus 6 months, p = 0.17). Patients with LCNEC and BM have a poor prognosis, particularly when synchronous BM are present. Prospective trials are required to define optimal therapeutic algorithms.

## Introduction

Large cell neuroendocrine carcinoma of the lung (LCNEC) is a rare and highly aggressive type of lung cancer, representing approximately 3% of primary lung malignancies [1, 2]. The disease is associated with an abysmal prognosis, with more than half of patients presenting with metastatic, i.e. stage IV disease. Currently available literature shows a median survival time of 9 months and a 2-year survival rate below 30% across all disease stages; in metastatic disease, these figures are estimated at 5 months and 9%, respectively [3].

Histologically, LCNEC represents a unique tumor entity, exhibiting histopathologic features of both small- (SCLC) and non-small cell lung cancer (NSCLC). In detail, a large cell phenotype with abundant cytoplasm, neuroendocrine morphology, and expression of at least one of three neuroendocrine markers (CD56, chromogranin A or synaptophysin) are required for diagnosis [4]. On the molecular level, genomic profiling has revealed two molecular subtypes of LCNEC [5]. These can be characterized as SCLC-like and NSCLC-like, with the SCLC-like subtype exhibiting co-mutations or loss of TP53 and RB1, whereas the NSCLC-like subtype is defined by wild type TP53 and RB1 and presence of mutations typical for NSCLC, such as STK11, KRAS and KEAP [6]. In the 2015 WHO classification of pulmonary neuroendocrine neoplasms, LCNEC was grouped together with SCLC and low- and intermediate-grade carcinoid tumors [7]. This nomenclature has remained largely unchanged in the latest 2021 WHO classification of thoracic malignancies [8].

According to larger series, LCNEC shows a higher incidence of brain metastasis (BM) with 19.2%, compared to both SCLC and NSCLC (16.7% and 13%, respectively), with BM being present in up to 35% of LCNEC patients with stage IV disease [3, 9]. Consequently, screening for BM with cranial imaging after initial diagnosis of LCNEC – in line with the algorithm for diagnostic workup in SCLC – is routinely carried out according to current guidelines, such us the EANO-ESMO guideline, with cranial magnetic resonance imaging (MRI) being considered the gold standard [10]. As very limited data are available regarding the clinical course and treatment outcome of patients with LCNEC and BM, management decisions in this setting are largely based on clinical experience. Some data indicate superiority of stereotactic radiosurgery (SRS), i.e. Gamma Knife treatment, in comparison to whole brain radiation therapy (WBRT) [11]. Small retrospective studies have investigated the role of prophylactic cranial irradiation in LCNEC; however, there is no consensus on this topic to date [12].

Patients diagnosed with brain metastases between 1990 and 2022 (regardless of primary tumor localization and histology) who were treated at the Medical University of Vienna, a tertiary referral center for patients with thoracic malignancies, have been included in the Vienna Brain Metastasis Registry. This dataset provides valuable data specific to BM, such as size and number, associated symptoms, and implemented treatment modalities, documented chronologically from the time of initial diagnosis [13]. The current analysis aimed to characterize the distinct cohort of patients with LCNEC and BM.

## Methods

### Patient selection

This retrospective analysis investigated patients with a histologically verified diagnosis of LCNEC and established BM who were treated at the Medical University of Vienna between January 1990 and May 2022. Patient data were extracted from the Vienna Brain Metastasis Registry. Patients with mixed histologic phenotypes (e.g. LCNEC and adenocarcinoma) were excluded. General clinical and BM-specific data from the time of diagnosis of LCNEC as well as treatment-related data were retrospectively assessed from routine medical records and anonymized for further analysis. In addition, survival data from the SCLC BM cohort were extracted and served as comparison cohort [14]. This investigation has been approved by the local ethical board of the Medical University of Vienna (EK No 1051/2022).

### Patient characteristics

Baseline patient characteristics including sex, age, smoking status, TNM staging at initial presentation, time to BM, Karnofsky performance status index (at BM diagnosis), as well as graded prognostic assessment (GPA) and diagnosis-specific GPA (DS-GPA) scores for NSCLC were retrieved from the Vienna Brain Metastasis Registry based on electronic medical records. Furthermore, information about treatment modalities (neurosurgical resection, radiation therapy, systemic chemotherapy or best supportive care) with focus on treatment of BM was extracted. Finally, an assessment of overall survival (OS) after LCNEC diagnosis and after BM diagnosis was carried out. The cause of death was evaluated in all patients for whom such data were present.

### Brain metastasis characteristics

BM were diagnosed with computed tomography (CT) or MRI. We recorded the diameter (cm), localization, and number of cranial lesions. Symptoms attributed to BM were documented and grouped into the following categories: (1) neurological deficits such as aphasia, sensorimotor dysfunctions, vertigo, impaired vision, and cognitive impairment; (2) signs of increased intracranial pressure such as emesis and headache, and (3) epileptic seizures, including focal and generalized seizures.

### Statistical analysis

The date of the histological verification of the primary tumor was determined as the date of LCNEC diagnosis. BM and LCNEC were defined as “synchronous” when both were diagnosed within 30 days; all other cases were defined as “metachronous”. In patients who did not present with initial cerebral metastases, the time to development of BM was assessed. Overall survival times (OS) after initial diagnosis of LCNEC and after diagnosis of BM were calculated; furthermore, OS in first-line SRS vs. other first-line BM treatment groups and OS per GPA and DS-GPA classes was compared [15, 16].

All statistical analyses were performed using SPSS version 28 (SPSS Inc., Chicago, USA). Metric variables were presented with medians and range (minimum/maximum). For categorical variables, we show absolute and relative (percentage) frequency. Survival times were estimated using the Kaplan Meier method and presented with 95% confidence intervals (CI). For comparison of survival groups, a log-rank test was performed, where p-values below 0.05 were considered statistically significant.

## Results

### Patient characteristics

At the cut-off date in May 2022, the Vienna Brain Metastasis Registry contained data from 6083 patients. In total, 3138 (51.5%) had a diagnosis of lung cancer, of whom 52 individuals had histologically verified LCNEC (0.09% of the entire cohort and 1.6% of all patients with lung malignancies). Within this group, 23 (44.2%) patients were female and 29 (55.8%) were male. Smoking status was documented in 44/52 patients; 36 patients (69.2%) were confirmed smokers, and 8 patients were listed as non-smokers. Detailed patient characteristics are shown in Table 1.

**Table 1:** Baseline characteristics of patients with LCNEC of the lung and brain metastases

Patient characteristics	Total number of patients (n = 52)
Sex, n (%)	
Female	23 (44.2%)
Male	29 (55.8%)
Age at diagnosis of LNCEC in years, median (range)	59.1 (46–82)
Age at diagnosis of BM in years, median (range)	60.1 (46–87)
Smoking status, n (%)	
Yes	36 (69.2%)
No	8 (15.4%)
No information	8 (15.4%)
Stage IV at diagnosis of LCNEC	
Yes	29
No	23
Localisation of BM, number of patients	
Frontal lobe	16
Parietal lobe	7
Temporal lobe	8
Occipital lobe	10
Cerebellum	15
Number of BM	
1	27
2	6
3	7
>3	12

In addition, patients with a diagnosis of SCLC and BM were extracted as survival control group (n = 553, 9% of the entire cohort and 17.6% of all patients with lung malignancies).

### Clinical characteristics at diagnosis of LCNEC

The median age at diagnosis of LCNEC was 59.1 years, with a range of 46 to 82 years. A total of 29 patients (55.7%) had stage IV disease at initial diagnosis of LCNEC, and 21 (40.3%) had synchronous cerebral metastases. For the 31 patients who did not present with cerebral metastases initially, the median time to development of BM was 13 months (range 4-165). Other sites of metastatic disease in the cohort were liver (n = 11), bone (n = 10), adrenal glands (n = 6), and kidney (n = 2).

Regarding therapy of extracranial disease, surgical resection of the primary tumor (either lobectomy or pneumonectomy) was carried out in 24 (46.1%) patients, of whom 3 patients underwent adjuvant radiotherapy. Furthermore, 34 patients received either adjuvant (n = 14) or palliative (n = 20) chemotherapy. Documentation regarding specific chemotherapy protocols was available in 24 patients, all but one (n = 23) of whom received platin-based treatments; the only exception was a single patient who was given a combination of temozolomide and capecitabine as palliative chemotherapy.

### Clinical characteristics at diagnosis of BM

The median age at diagnosis of BM was 60.1 years (range 46–87). Most (n = 27, 52%) patients presented with a single BM, n = 6 (11.5%) with 2 BM, and n = 7 (13.4%) with 3 BM. The remaining patients (n = 12, 23%) presented with 4 or more lesions. Notably, one patient had 12 metastases spread in a diffuse pattern. The median lesion size was 2.5 cm (range 0.3-6 cm), and 14 individuals had lesions measuring more than 3 cm. In the subgroup of 35 patients with unilateral BM, the left hemisphere was slightly more often affected than the right, with 20 patients (57.4%) showing left-sided lesions. Frontal localization of the brain lesions occurred most often (n = 16), followed by cerebellar (n = 15), occipital (n = 10), temporal (n = 8), and parietal (n = 7).

The median Karnofsky performance status index at diagnosis of BM was 80%. Regarding GPA score, n = 3 (5.7%) were allocated to class I, n = 3 (5.7%) to class II, n = 36 (69.2%) to class III, and n = 10 (19.2%) to class IV. As per DS-GPA score, the class distribution was n = 3 (5.7%) in class I, n = 13 (25%) in class II, n = 26 (50%) in class III, and n = 10 (19.2%) in class IV.

The majority of patients (59.6%, n = 31) showed neurologic symptoms associated with newly detected BM, which were categorized as focal neurological deficits (n = 18), signs of increased intracranial pressure (n = 14), or epileptic seizures (n = 5), with 6 patients presenting with symptoms overlapping between these categories. An additional n = 9 patients developed neurologic symptoms during later course of disease, of which n = 4 showed signs of increased intracranial pressure, n = 6 developed some form of neurologic deficit, and seizures being documented in one individual.

### BM-specific treatment and survival

**Fig. 1:**
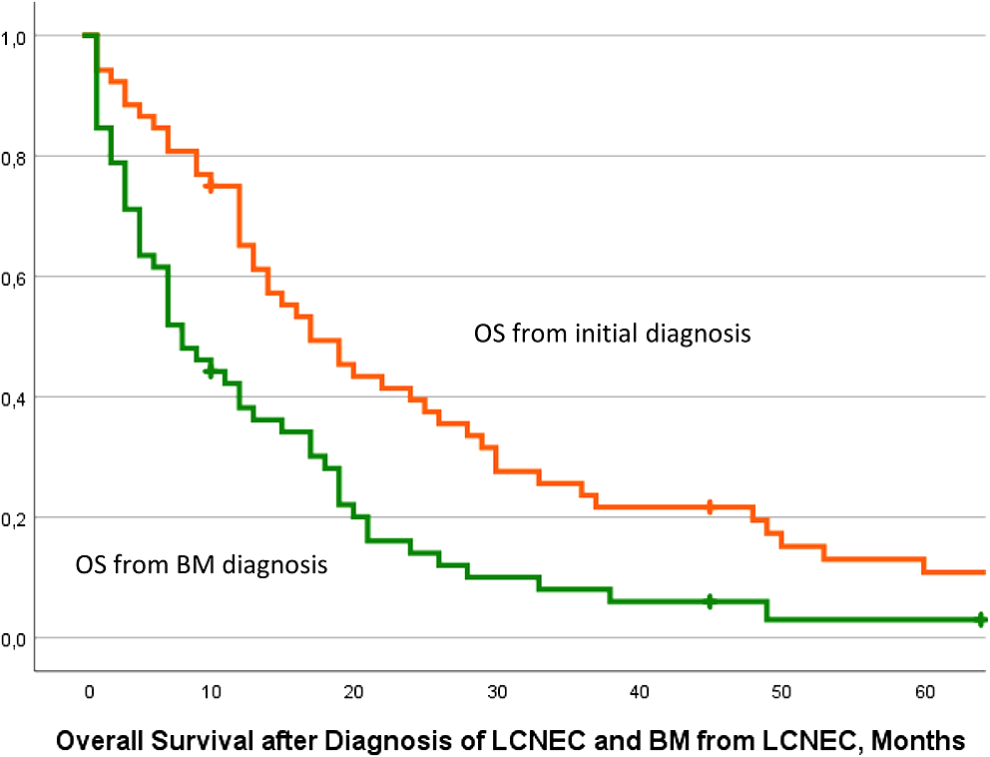
Kaplan Meier survival curves for estimated overall survival in patients with LCNEC and BM

The median overall survival (OS) of LCNEC patients was 16 months (range 1-192, 95% CI 10.2–21.7), and the median OS from BM diagnosis survival was 7 months (range 1–63, 95% CI 3.8–10.1), as shown in the Kaplan-Meier survival curves in Fig. 1.

Median OS decreased according to the GPA classes with 18 months, 16 months, 6 months, and 2 months for classes I-IV, respectively, however, no statistically significant difference was detected (p = 0.322). Similar results were obtained with the DS-GPA.

**Fig. 2:**
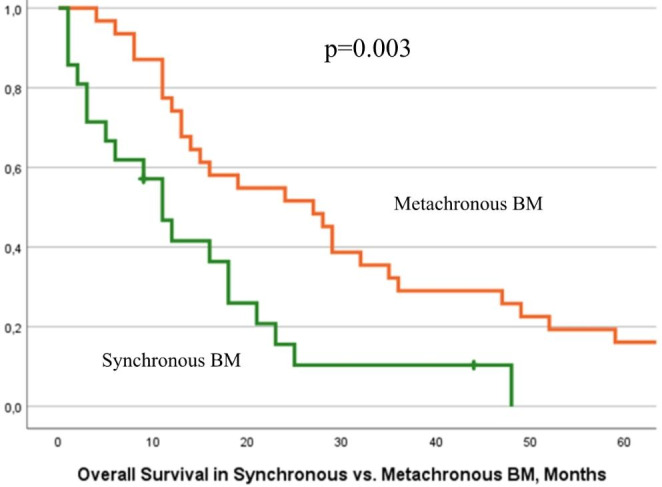
Kaplan Meier survival curves for estimated overall survival from LCNEC diagnosis for synchronous vs. metachronous BM

OS after LCNEC diagnosis was shown to be significantly reduced in synchronous vs. metachronous brain metastases (Fig. 2).

In detail, the median OS from initial LCNEC diagnosis of patients with synchronous metastases was 11 months (range 1–48, 95%CI 6.82–15.17), compared to 27 months in patients with metachronous cerebral involvement (range 4-192, 95% CI 15.18–38.81), with a p-value of 0.003. There was no statistically significant difference in OS after BM diagnosis in patients presenting with single vs. multiple BM; i.e. median OS 9 vs. 6 months, ranges 1–63 vs. 1–44, 95% CI 2.39–15.60 vs. 5.02–6.97, p = 0.66.

Regarding first-line treatment after diagnosis of BM, all but 3 patients received local or regional therapy with either neurosurgical resection of BM (n = 13, 25%), WBRT (n = 19, 36.53%), or SRS (n = 25, 48.07%) – alone or in combination, with one patient treated with all three methods. Of the remaining 3 patients, one was treated with systemic chemotherapy alone after BM diagnosis, and two received best supportive care. A log-rank test was performed to compare survival after BM diagnosis between patients who received initial treatment with SRS and/or neurosurgical resection (both representing an aimed anatomical approach) vs. patients who underwent WBRT, which showed no statistically significant difference with a median OS after BM diagnosis of 7 months for SRS and/or neurosurgical resection (range 1–63, 95% CI 3.04–10.95) vs. 6 months for WBRT (range 1–19, 95% CI 0.97–11.02), p = 0.135.

The specific cause of death could be ascertained in 21 patients, with a clear correlation between development and/or progression of BM and death evident in 9 patients. Other causes of death included overall tumor progression and/or cachexia (n = 9), sepsis (n = 1), peritonitis (n = 1), and multiorgan failure following resection of the primary tumor (n = 1).

**Fig. 3:**
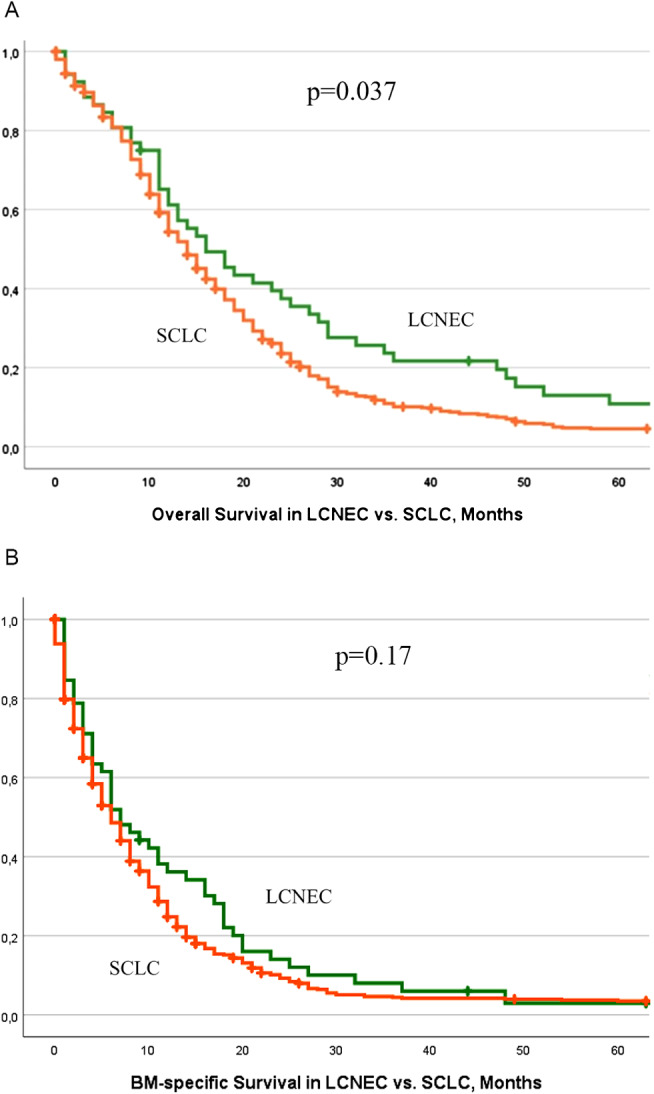
Kaplan Meier survival curves in patients with LCNEC and BM vs. patients with SCLC and BM. (**A**) Overall survival after LCNEC diagnosis. (**B**) Overall survival after BM diagnosis

Finally, a comparison of survival between LCNEC and SCLC patients from our Registry was carried out, with a total of 553 SCLC patients with BM identified. Survival after initial diagnosis was significantly shorter in SCLC (p = 0.037). In detail, median OS measured 14 months in SCLC (range 0-269, 95% CI 12.74–15.25) vs. 16 months in LCNEC (range 1-192, 95% CI 10.21–21.78). However, there was no relevant difference in median OS from BM diagnosis (SCLC: mOS 6 months, range 0-264, 95% CI 5.11–6.88; LCNEC: mOS 7 months, range 0–63, 95% CI 3.86–10.13; p = 0.17) (Fig. 3).

The SCLC cohort in our Brain Metastasis Registry has been described in greater detail by Steindl et al. and shows comparable age at BM diagnosis (median 61 years), number of BM and ratio of SRS, WBRT, and surgical resection as local treatments as compared to our LCNEC cohort [17].

## Discussion

LCNEC poses a largely unexplored area in oncology, and given the poor prognosis and lack of standardized guidelines, there is a clear unmet clinical need. Particularly as LCNEC has been shown to substantially differ from its counterpart SCLC in terms of molecular biology and clinical behavior, distinct prospective data are required in order to further separate the two entities and elaborate a LCNEC-specific therapeutic algorithm [5]. Accounting for a relatively small fraction of the entire LCNEC population, patients with BM represent an even less studied cohort and, therefore, all available data on this population are highly valuable.

Our study was conducted using data from the Vienna Brain Metastasis Registry, which contains more than 6,000 patients with brain metastases of diverse tumor types treated at the Medical University of Vienna, a large tertiary center for cancer patients. Importantly, the dataset includes not only general clinical features, treatment and long-term survival of cancer patients but also extensive BM-specific data such as localization, distribution and associated symptoms. This allows the unique opportunity to carry out detailed analyses of neurological presentation and BM-specific characteristics paired with therapeutic outcomes.

Only 52 patients in our BM-dataset, i.e. 0.09% of the overall collective, had a diagnosis of LCNEC, again underlining the rarity of this diagnosis. In comparison, a total of 553 SCLC patients have been documented in the same dataset. As expected, the identified LCNEC patients had a limited survival time of median 7 months following the diagnosis of BM and 16 months following the initial diagnosis of LCNEC, respectively. While there are only vague data regarding BM-specific OS in the literature, these numbers compare to a large US National Cancer Database analysis of LCNEC BM patients treated with SRS or WBRT, reporting a median OS of 11 and 6 months for both cohorts respectively [11].

A large SEER database study that included a total of more than 1,500 LCNEC cases found a median OS of 7 months for patients with BM at initial diagnosis and a median OS of 9 months for the entire cohort at all stages. While our mixed cohort of patients with BM at initial diagnosis and development of BM later on during disease is not directly comparable, the reported median OS of 16 months in this small single-center analysis appears relatively long [3].

In terms of demographic structure, our cohort showed a slight male predominance (55.8%) and incidence mainly in the sixth and seventh decades, which correlates with previous data [3, 12]. Most patients had a high symptom burden – both neurologic symptoms as well as in view of the overall performance status. We therefore recommend that early palliative care consisting of symptom management and psychosocial support should be integrated in the treatment strategy from the point of initial diagnosis. This particularly applies to the subgroup of patients presenting with synchronous BM, who had a significantly worse mOS in our analysis (p = 0.003), and is also supported by a Cochrane analysis which has shown overall benefits in health-related quality of life in adults with advanced cancer [18]. In addition, assessment of the GPA and DS-GPA scores [15, 16] could be helpful to identify prognostic subgroups, as the classes roughly allowed stratification of OS in our patient population, but not to a statistically significant extent, which could possibly be due to the small number of patients in our dataset. Intracranial presentation of metastases in terms of localization, size and distribution did not reveal any specific patterns of interest.

As LCNEC in general has a higher incidence of BM compared with SCLC, and early detection appears crucial, we support screening for BM with either CT or MRI (preferably the latter due to higher image quality) in every patient with newly diagnosed LCNEC [3, 9, 10]. Given the histopathological, clinical, and therapeutic parallels, we also compared the LCNEC cohort to SCLC BM patients in our database and found a slight numerical but statistically significant longer OS from initial diagnosis for LCNEC vs. SCLC patients, while from BM diagnosis comparable. This is mostly in line with the general impression that advanced LCNEC present with a comparable to slightly superior prognosis than SCLC [3, 9].

Regarding therapy for LCNEC, resection and radiotherapy are the cornerstones of treatment in localized disease [1, 19], with adjuvant chemotherapy having been shown to improve outcome [20–22]. In metastatic / non-resectable disease, the first-line approach to chemotherapy consists of a platin-etoposide doublet, similarly to first-line therapy in SCLC, but currently available recommendations are based on retrospective trials with relatively small sample sizes [4, 23, 24].

However, in contrast to SCLC and non-LCNEC NSCLC, the role of systemic therapies with potential CNS-activity, such as immune checkpoint inhibitors (ICIs) and small molecules, remains undefined. A large retrospective study by Komiya et al. showed an improvement in 12- und 18-month survival in ICI recipients with LCNEC [25], but due to the lack of positive prospective data, no ICIs are currently approved for treatment of LCNEC. Tyrosine kinase inhibitors (TKIs) for targetable driver mutations, in line with oncogene-addicted NSCLC, represent a further area of interest in the treatment of LCNEC. Several case studies have reported response to therapy in LCNEC with detected targetable mutations, e.g. EGFR [26, 27] or ALK [28, 29]. These data are, however, insufficient to provide any overall treatment recommendations and the frequency of these mutations is low. While molecular classifications [5, 6] currently remain of academic interest, insights into the underlying genetics of LCNEC may aid in developing future treatment strategies in these distinct subtypes.

Thus, local therapy is the mainstay of BM-management in LCNEC, and was conducted in all but 3 patients in our cohort. WBRT and SRS constituted the most frequently applied strategies. A relatively large multicenter series from Japan reported satisfactory control of neurologic status after SRS, and a study by Wegner et al. even suggested a survival benefit of upfront SRS treatment [11, 30]. In our collective, there was no statistical survival difference between patients treated up-front with SRS vs. other first-line local treatment (8 vs. 6 months, p = 0.257). As data remains controversial, we recommend that patients be referred to facilities which offer all possible treatment modalities.

The study has several limitations. Firstly, it is a retrospective analysis of a relatively small cohort of 52 patients. Partly due to the large time span of the data within the database, certain pieces of clinical information could not be precisely ascertained, such as exact chemotherapy protocols or systemic therapy lines; it should however be noted that a time-dependent evolution of treatment and survival patterns in the overall BM cohort at our center was previously published [14]. In most patients, the exact cause of death was not documented, as they were not hospitalized at our facility at the time of death. Karnofsky performance status was assessed at the time of diagnosis of BM, but not at the time of initial diagnosis of LCNEC. Furthermore, genetic analysis of driver mutations as well as immunohistochemistry for PD-L1 status was too scarce as to be discussed here.

In conclusion, BM in LCNEC remain a crucial event regarding prognosis, particularly if patients present with synchronous BM. Prospective data and molecular characterization appear necessary to move forward from expert opinion-driven decisions to an evidence-based approach in the treatment of BM in LCNEC.
